# Maternal Odor Exposure Modulates Acceptance of a Bitter Taste in Newborn and Infant Rats

**DOI:** 10.3389/fpsyg.2018.01327

**Published:** 2018-07-31

**Authors:** María C. Ifrán, Andrea B. Suárez, Ricardo M. Pautassi, Giselle V. Kamenetzky

**Affiliations:** ^1^Instituto de Investigaciones Médicas Alfredo Lanari, IDIM-CONICET, Universidad de Buenos Aires, Buenos Aires, Argentina; ^2^Centro de Altos Estudios en Ciencias Humanas y de la Salud – Universidad Abierta Interamericana, Buenos Aires, Argentina; ^3^Instituto de Investigación Médica Mercedes y Martín Ferreyra, INIMEC-CONICET-UNC, Córdoba, Argentina

**Keywords:** odor, taste, ontogeny, bitter, mother, rats

## Abstract

The acceptance of bitter, aversive, substances during early life is enhanced by stimulation with familiar, pre-exposed odors. Newborn rats exhibited heightened grasp responses toward an artificial nipple dispensing quinine, and drank more of this bitter solution, if concurrently stimulated with a lemon odor they had been exposed to shortly after birth. It yet unknown, however, if odors made familiar via normative developmental milestones also acquire modulatory influence upon seeking and intake of basic tastants. The current study assessed the influence of exposure to maternal odor on intake and grasp responses toward a surrogate nipple providing quinine, in 3-day (Experiment 1) or 12-day (Experiment 2) old, Wistar rat pups. The results revealed enhanced seeking and intake of the bitter solution, but not of water, in animals tested in the presence of the mother (and hence exposed to its odor cues), at both ages, compared to counterparts given either no explicit odor stimulation or stimulation to the odor of an unrelated dam. These results, obtained with a biologically relevant odor, are consistent with those previously found with a neutral, arbitrary odor. It seems that during the early stages of development, familiar odors regulate the acceptance of non-palatable, otherwise rejected, flavors.

## Introduction

Various neuroendocrine mechanisms are in place to keep the caregiver in close proximity during the early development of altricial species, which in turn increases access to food, protection from predators, and warmth (Upton and Sullivan, 2010). Attachment behavior is regulated by sensorial stimuli such as sight and olfaction (Morrow-Tesch and McGlone, 1990; Janzen et al., 1999; González-Mariscal and Poindron, 2002). Birds learn to identify the caretaker through visual stimuli, briefly after birth. However, in mammals, olfaction has a more predominant role (Nowak et al., 2011).

In humans, olfactory stimuli are essential in the acquisition of early learning and induce approach toward the maternal breast, thus helping deploy intake behaviors (Mennella et al., 2016). During the last trimester of gestation fetuses ingest the amniotic fluid (Lipchock et al., 2011), detecting odors and tastes carrying individualized sensory information from the mother. Re-exposure to these stimuli, for instance during breastfeeding, impacts diet choice during postnatal life (Mennella et al., 2001; Lipchock et al., 2011). Taste is essential to ingestive behavior and is a product of the gustatory and olfactory systems working together (Auvray and Spence, 2008).

Infants from a variety of species usually reject bitter food. There seems to be, however, a sensitive period in which early experiences with bitter tastes predispose them to accept this, otherwise unpalatable, taste (Beauchamp and Mennella, 2009). Human babies accept bitter tastes during the first 4 months, but reject them afterwards (Mennella et al., 1996; Beauchamp and Mennella, 2011). This sensitive period would allow culture-dependent shifts in innate reactivity to food and flavors, which are essential for establishing long-term healthy eating habits (Trabulsi and Mennella, 2012).

The newborn rat is an appropriate animal model for the study of gustatory learning. Rat pups develop the sense of sight and hearing by the third week of life (Sullivan and Holman, 2010), therefore they depend on their mother’s odor to monitor the localization of the nest and the caregiver (Hofer, 2005). The absence of that odor triggers locomotion and distress calls (i.e., ultrasonic vocalizations) that increase the likelihood of reuniting with the dam. Thus, olfactory learning can help discriminate changes in odors resulting from the fluctuating dietary patterns from the mother (Landers and Sullivan, 2012). The evolutionary relevance of olfactory learning apparently favored the emergence of a sensitive period (i.e., first 10 postnatal days, PND) in which familiar odors are preferred, even after their association with a moderate aversive situation (Sullivan et al., 2000). Even neutral, arbitrary, odors can significantly control the pup’s behavior if made familiar by odor pre-exposure. For instance, exposure to a lemon odor, made familiar by pre-exposure, increased responsiveness toward a surrogate nipple in neonatal rats, compared to rats that did not have experience with this odor (Miller and Spear, 2008, 2009, 2010).

Neonate rats discriminate between different tastants, as shown by differential suckling behavior toward saline and quinine, in the first hours of life (Nizhnikov et al., 2002). Yet there are few studies assessing the interaction of taste and smell at this early age. Kamenetzky et al. (2014) gave rats a brief exposure to a lemon odor (or no exposure, control group), shortly after birth, and then tested intake from and seeking of a lemon-odor scented, surrogate, nipple dispensing 0.1% quinine. Pups that had been pre-exposed to the lemon odor exhibited, when compared to controls, significantly greater seeking of the nipple and quinine intake than controls. This effect did not occur when the nipple provided saccharin. This intriguing experiment suggests that familiar odors can enhance the acceptance of aversive solutions.

The present study assessed seeking and drinking of a bitter solution in 3-day old or 12-day old rats, in the presence of a scent extracted from their own mother or from another, unrelated, mother. We aimed at assessing the generality of the pre-exposure effect described in our previous work. We used, instead of an arbitrary (e.g., lemon) odor, a biologically relevant odor, made familiar through the natural history of interactions and shared milieu of the dam/pup dyad. Our hypothesis was that the explicit stimulation with the maternal odor, yet not the odor of an unrelated dam, would increase quinine seeking and intake.

## Materials and Methods

### Subjects

Ninety-six, 3 or 12 day-old male and female pups, rats were employed. These animals were derived from 13 dams, mated at the vivarium of Instituto de Investigaciones Médicas Alfredo Lanari (IDIM-CONICET, Argentina). Thirty-four and sixty-two animals were employed in Experiments 1 and 2, respectively. Pups were born by natural delivery, the day of parturition was considered PND 0, and litters had *ad libitum* access to water and food (Cooperación Co., Buenos Aires, Argentina). The vivarium had a 12 h/12 h light/dark cycle, with lights on at 7 am, and controlled temperature (22°C) and humidity. Rats used in these experiments were maintained and treated in accordance with the guidelines for animal care and use established by the National Institutes of Health (National Research Council, 1996). The protocol was approved by the Animal Care and Use Committee of IDIM-CONICET of Instituto de Investigaciones Médicas Alfredo Lanari (Approval number 0602-17).

### Experimental Designs

Experiment 1 measured seeking and intake of quinine in 3-day old rats via a two-group design (Experimental and Control, 18 and 16 animals in each group, respectively). Experiment 2 assessed intake of quinine or water in 12-day old rats. In Experiment 2, the pups were distributed in four groups, as a function of the solution delivered via the nipple (water or quinine), and depending on whether the test was conducted in the presence of the pup’s dam or in the presence of an unrelated dam. Each group was composed by 15–16 animals.

### Surrogate Nipple

The surrogate nipple used in Experiment 1 was made from rubber latex (AMACO rubber latex, Indianapolis, IN, United States) and molded into a conical form to measure 12 mm long with a rounded tip measuring 1 mm in diameter and the base measuring 2.5 mm in diameter. The base of the surrogate nipple was attached to the end of an angled dental probe to facilitate presentation by the experimenter (Petrov et al., 1997). Polyethylene tubing (Clay Adams, Sparks, MD, United States) extended throughout the length of the nipple and attached to a syringe containing the solutions. The syringe had a hole on the wall that generated a hydraulic system. When the oral cavity of the pup contacted with the latex and the tip was pressed by the mouth, a negative pressure was generated that allowed voluntarily intake. The subject was clamped in a semi-supine posture into a “vest” made from ultra-thin, elastic rubber. This light restraint prevented righting attempts but did not otherwise produce discomfort nor hinder movement. The pups were kept, before the test, in a controlled heat chamber (Simen, Buenos Aires, Argentina) maintained at 35°C (PND3) or 28°C (PND12).

### Procedure

On the morning of PND 3 (Experiment 1) or PDN12 (Experiment 2), the dam was separated from their pups and anesthetized with a combination of ketamine (40 mg/kg) and xylazine (5 mg/kg). The pups were placed into the heating chamber for 15 min. Each pup was gently stimulated in the urogenital region with cotton to induce urination or defecation and subsequently weighted in a precision scale.

Experiment 1 followed an adapted version of the protocol described in Kamenetzky et al. (2014). Briefly, each pup was equipped with a vest and attached to a tempered mirror. The test involved a 6-min stimulation with a 0.1% quinine solution (i.e., 100 mg quinine in 100 ml of distilled water), dispended through the artificial nipple. The tip of the nipple was gently applied on the perioral area, in the presence (Experimental Group) or absence (Control Group) of the mother. The anesthetized mother was placed 2 cm away from the nose of the pup.

In Experiment 2 the pups, 12-day old rats, were separated from the dam and intraorally cannulated, as described in Pautassi et al. (2008). The intraoral devices (PE 10 polyethylene tubing, 5 cm length) were made using a heat source to flatten one of the ends. A dental needle, attached to the non-flanged end, was used to place the cannula in the middle portion of the mucosa, with the flattened end inside. This procedure requires ±8 s per subject and does not induce major stress (Spear et al., 1989). After the cannulation, the pups were group-housed into a chamber kept warm (i.e., 28°C) until the time of the test.

Animals were then weighted, stimulated in the urogenital region and placed into the heated chamber for 10 min. The anesthetized mother (either the pup’s dam or an unrelated dam) was also placed into the chamber, in close proximity to the pups. The inclusion of pups exposed to an anesthetized nursing mother controlled the effects of factors other than the exposure to the mom’s odor (e.g., heat source, social contact). In order to assess the specificity of the type of taste associated to the mother’s odor, another condition included the delivery of water.

To prevent pups from suckling from the nipples, the dams were wrapped in a Clingfilm envelope, which had an opening in the back to allow dissemination of the mother’s odor but prevented the pups from coming in contact with the nipples. Animals were weighted and placed into the heated chamber for 10 min. Each intraoral cannula was attached to a PE50 length, which in turn was connected to an infusion pump (APEMA, Buenos Aires, Argentina), equipped with four Prexajet 5-ml syringes. The pump delivered water or quinine at a continuous rate, and the volume was adjusted to deliver 2.5% of the pup’s body weight. This procedure combines forced administration of the solution with voluntary intake, because the rats can actively reject the solution infused by emitting aversive taste responses (e.g., chin rubbing, passive drips, etc.). Body weights were registered at the end of each test, and the heated chamber was cleaned with a damp cloth.

The percentage of body weight gained [(post-weight–pre-weight)/pre-weight] × 100] was calculated in both Experiments, whereas total time of grasp, number of grasp, latency to grasp and mean grasp duration toward the artificial nipple were recorded in Experiment 1 only. The behavioral coding was analyzed by two independent observers. The inter-rater reliability was >85% for all the behaviors, and was calculated was follows [Agreements/(Agreements + Disagreements)]^∗^100; for the frequency, and (Shorter amount of time/Longer amount of time)^∗^100, for duration.

### Statistical Analysis

The body weight gained and the behavioral responses emitted during Experiment 1 were analyzed via Analysis of Variance (ANOVA). In Experiment 2 we used two-way ANOVA guided by our priory hypotheses, we used planned comparisons to analyze intake scores, separately for water and quinine, between pups tested in the presence of the pup’s dam or in the presence of an unrelated dam. Preliminary analysis indicated that, across variables, sex did not exert significant main effects nor significantly interacted with the remaining variables. Therefore, data were collapsed across sex. The alpha level was set at 5%.

## Results

**FIGURE 1:**
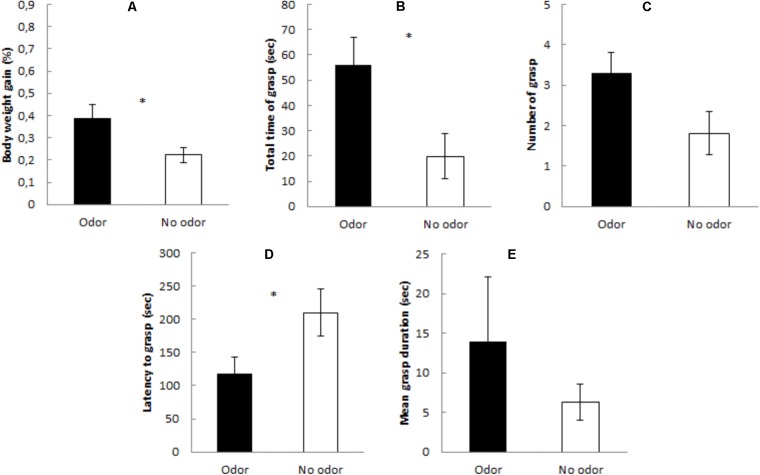
Mean percentage (±SE) of **(A)** body weight gain, **(B)** total time of grasp, **(C)** number of grasp, **(D)** latency to grasp, and **(E)** mean grasp duration during the 6-min presentation of the artificial nipple containing quinine in the presence of the mother (black bars – Odor condition) or in the absence of the mother (white bars – No odor condition). ^∗^Indicates *p* < 0.05.

**Figure 1** shows percentage of body weight gained during the test, total time of grasp, number of grasps, mean grasp duration, and latency to grasp, in Experiment 1. It seems that the presence of the maternal odor significantly reduced the latency to grasp the nipple, increased the time attached to it and heightened quinine intake. These impressions were confirmed by the statistical analyses. Subjects evaluated in the presence of the mother exhibited, when compared to controls assessed in isolation, significant increases in body weight gained [*F*(1,32) = 4.98, *p* < 0.03] and in total time of grasp [*F*(1,32) = 6.58, *p* < 0.01]; and a significant decrease in the latency to grasp the nipple [*F*(1,32) = 4.53, *p* < 0.04]. Likewise, the ANOVA for number of grasp responses neared significance [*F*(1,32) = 3.78, *p* > 0.06]. Mean grasp duration was similar across groups [*F*(1,32) = 2.97, *p* > 0.09].

**FIGURE 2:**
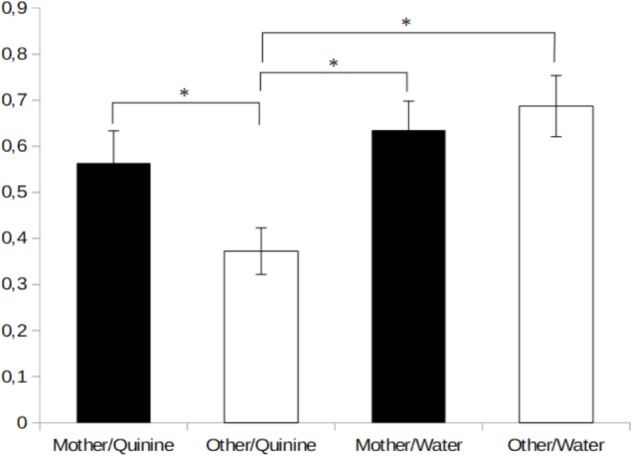
Mean percentage (±SE) of body weight gain during the 6-min administration of quinine or water in the presence of the mother’s odor or in the presence of another lactating mother. ^∗^Indicates *p* < 0.05.

The data gathered in Experiment 2 has been depicted in **Figure 2**. The analysis of ANOVA did not show interactions between the different groups. Therefore, guided by our priory hypotheses, we used planned comparisons to analyze intake scores. The animals stimulated with quinine in the presence of the odor of an unrelated dam, but not those tested in the presence of their own dam, rejected, for the most part, the quinine solution. Planned comparisons indicated that quinine intake in the group tested in the presence of their own dam was significantly higher compared to the rest of the groups, *F*_3,61_ = 4.83, *p* < 0.005. Pups stimulated with the odor of their own mother drank as much quinine as water (*p* > 0.05).

## Discussion

Olfactory cues produced by the dam regulate the attachment of pups (Sullivan and Holman, 2010). It is possible that maternal odors regulate other behavioral domains, yet the information on this topic has been scarce. The present study stimulated pups with a bitter solution, delivered through an artificial nipple at PND 3 (Experiment 1), or through intraoral administration at PND 12 (Experiment 2). The odor of the familiar significantly enhanced, when compared to control groups that were evaluated in isolation (Experiment 1) or in the presence of non-familiar dam (Experiment 2), seeking and intake of this bitter, normally aversive, solution.

These results suggest that exposure to the stimuli present in the nest are sufficient to overcome the innate disposition to reject aversive solutions, which was likely inherited due to the association of bitter tastes with poisons and malaise. Experiment 1, however, has the caveat of testing controls in isolation. It can be proposed that greater quinine intake, as found in the “dam group,” is merely a side-effect of the additional warmth or social contact provided by the mother. Experiment 2, however, tested all pups in close proximity with an adult female, yet those tested in the presence of an unrelated dam rejected quinine (i.e., the level of quinine intake was lower than the level of water intake in peers tested in similar conditions) and drank significantly less of this bitter solution quinine than pups tested in the presence of their own dam. Therefore, it seems that only the odor of a familiar adult female (i.e., the dam) heightens quinine intake. The mother’s odor does not increase water consumption, further pinpointing the specificity of the phenomenon under analysis. The pups stimulated with water drank as much of that fluid regardless the presence of their own mother or the presence of another, unrelated mother. The data is consistent with, and generalize, previous results in which exposure to a lemon odor increased responses to an artificial nipple dispensing a 0.1% solution of quinine, but only when the lemon odor had been pre-exposed immediately after birth. A possible limitation of the present study is, however, that the experiments were conducted at different ages. On the positive side, this suggests that, in the rat, the ability of maternal odor to inhibit the deployment of aversive responses seems to last until the end of the second week of life.

These results relate to previous evidence suggesting that the presence of the mother decreases the offspring response to aversive stimulation. More in detail, during a sensitive period that lasts up to PND 10, approximately, the pups learn conditioned odor preferences even after pairing odors with an electric shock of moderate intensity (Sullivan et al., 2000). Furthermore, maternal odor exposure seems to decrease stress responses in rats’ 2-weeks old or older, a phenomenon known as *social buffering* (Gunnar and Sullivan, 2017).

Also related to the present results is that, due to nutritional reasons, some newborns are fed with a protein-hydrolysate formula that has a strong bitter taste (Beauchamp and Mennella, 2011). This formula is rejected by babies older than 4–5 months, yet it seems to be accepted by younger babies, who display normative weight gain curves after chronic feeding with the formula (Mennella et al., 2011). Also, babies that had been exposed to garlic (Mennella and Beauchamp, 1993) or alcohol (Mennella and Beauchamp, 1991), due to prior maternal consumption, also seem to increase acceptance of milk contaminated with these flavors. Unpublished data from our laboratory also showed that infant rats stimulated with an alcohol taste in the presence of maternal odor increased consumption responses toward alcohol. Altogether, these data and studies suggest that during the early stages of development there is a sensitive period for flavor learning, in which the presence of the maternal odor may increase responses to bitter compounds, otherwise rejected, or can endow neutral, arbitrary learned odors with the ability to increase acceptance of these compounds.

Human infants learn about flavor stimuli through the amniotic fluid and later through breast milk and through formula. This learning can affect the development of food choices, thus exerting long-term effects upon health (Trabulsi and Mennella, 2012). The present report represents progress toward the development of an animal model for analyzing the influence of factors that increase the intake of bitter-tasting foods.

## Author Contributions

All authors participated in the design, interpretation of the studies, and analysis of the data and review of the manuscript. MI, AS, and GK conducted the experiments and analyzed the data. MI, AS, RP, and GK wrote the manuscript.

## Conflict of Interest Statement

The authors declare that the research was conducted in the absence of any commercial or financial relationships that could be construed as a potential conflict of interest.
